# Effect of an online teaching module on midwives’ knowledge, attitude and practice regarding intrapartum ultrasound: A quasi-experimental approach

**DOI:** 10.18332/ejm/195498

**Published:** 2024-12-11

**Authors:** Bram Packet, Samešová Adela, Richter Jute

**Affiliations:** 1 Department of Development and Regeneration, Unit of Woman and Child, Catholic University of Leuven, Leuven, Belgium; 2 Department of Obstetrics and Gynecology, University Hospital Leuven, Leuven, Belgium; 3 The Institute for the Care of Mother and Child, Prague, Czech Republic

**Keywords:** childbirth, labor, delivery, ultrasound, education, midwifery

## Abstract

**INTRODUCTION:**

Intrapartum ultrasound (IPUS) allows for a more reliable and reproducible assessment of fetal head station and position during labor. This study aimed to investigate how an online education module on IPUS impacts midwives' knowledge, attitudes, and practices (KAP) regarding this topic.

**METHODS:**

Midwives working in the labor ward of the University Hospital of Leuven (Belgium) were invited to participate in an educational program on IPUS in April 2023. A baseline KAP survey was completed upon enrolment, followed by an online education module on the intrapartum sonographic assessment of head station and position. Afterwards, a second KAP survey was completed. Score were compared using a two-sided Wilcoxon signed-rank test. A p<0.05 was considered significant. Statistical analyses were conducted using SPSS (version 29.0.2.0).

**RESULTS:**

A total of 45 midwives were eligible for inclusion and invited to participate. From these, 46.7% (21/45) agreed to take part. Attitudes towards IPUS were positive, as most perceived it as safe, time-efficient, and beneficial for medical-decision making. However, only two midwives (9.1%) sometimes used IPUS themselves during the second stage, whereas none used it during the first stage. A significant improvement in knowledge scores was recorded after the online education module for both the sections on fetal head station (median score 0/5 to 2.75/5, p=0.01) and position (median score 2.5/5 to 3.5/5, p=0.04). No significant differences were observed in the overall attitude scores, as they remained overall positive (4.5/5 to 5/5, p=0.18).

**CONCLUSIONS:**

Although having little experience with IPUS themselves, most participating midwives perceive it as an acceptable, time-efficient, and safe imaging modality. A short online education module resulted in a significant improvement in their knowledge of these topics. Further implementation research is needed to investigate how the uptake of IPUS amongst midwives can be improved, and how this can improve overall labor care.

## INTRODUCTION

Ultrasound has become an indispensable imaging technique in the labor ward^1^. It is commonly used to check fetal presentation or aid fetal heart rate monitoring during labor. More recently, it has been shown to allow for the evaluation of fetal head position and station more accurately and reliably compared to digital vaginal examinations^2^. Moreover, parturient women prefer it, as it results in less pain and discomfort^3-5^.

Evaluating labor progress is a key responsibility of midwives. Early and accurate detection of labor dystocia, for instance because of fetal head mispositioning, could allow for targeted interventions possibly improving birth outcomes for the mother and neonate^6,7^. Seeing its availability in labor wards, non-invasive character, and safeness, ultrasound could be a useful point-of-care test for midwives to aid medical decision-making (e.g. regarding maternal positioning or need for manual rotation in case of occiput posterior presentation during the first or second stage). As with every ultrasound examination, accurate sonographic assessment of fetal head position and station is operator-dependent. Insufficient knowledge and skills can result in erroneous acquisitioning or interpretation of scan findings. This can result in delayed or inadequate decision-making, which can negatively affect patient care.

To our knowledge, no study has investigated midwives’ knowledge of intrapartum ultrasound or whether they perceive it as beneficial for parturient care. This is relevant, as it could inform the design of future implementation studies. The main objective of this study was to investigate midwives’ knowledge of, attitudes towards, and practice of intrapartum ultrasound. We also studied how a short online educational module on these topics influenced the latter.

## METHODS

### Study design and setting

This quasi-experimental study with a pre-post research design was conducted in the Department of Obstetrics and Gynecology, University Hospital of Leuven (Belgium). All midwives (n=45) working within the labor ward were invited to participate by an email sent out by the labor ward lead (JR) in April 2023. Enrolment was possible for 4 weeks, and those willing to participate were asked to give written informed consent.

### Participants

All midwives working in the labor ward were eligible for inclusion, without any restrictions regarding their age or years of clinical experience. The sample size was determined by convenience (i.e. willingness to participate).

### Knowledge, attitude, and practice (KAP) survey

A knowledge, attitude, and practice survey on intrapartum ultrasound was designed by the main study investigators (BP and AS) in collaboration with the senior investigator (JR). The survey consisted of 5 ‘knowledge’ questions on the sonographic assessment of fetal head position and station during labor, based on the 2018 International Society of Ultrasound in Obstetrics and Gynecology (ISUOG) guideline on these topics^2^. For fetal head station, a specific focus was on the angle of progression (AoP)^2^. Next, the survey asked about midwives’ attitudes regarding ultrasound use during labor (5 questions, i.e. whether they believed it was acceptable for patients or beneficial for their decision-making), and their current practice (i.e. whether they already used ultrasound themselves in certain clinical scenarios). For the knowledge section, respondents were able to judge each statement as ‘correct’ or ‘incorrect’, or indicate they had ‘no idea’. For the attitude and practice sections, statements could be judged either positively or negatively. For the final part of the KAP survey, participants were asked to indicate on a 10-point Likert scale how confident they felt performing intrapartum ultrasound themselves. It is worth noting that when the surveys were conducted, these tasks were typically performed by medical doctors working in the department. Hence, midwives were familiar with these techniques to a certain extent, though novices themselves.

Before implementation, the KAP survey was reviewed by four senior obstetricians with experience in intrapartum ultrasound, who were asked to provide feedback on the relevance of each question. It was also pilot-tested on a group of five first-year residents working in the department, who were like the midwives, novices in performing intrapartum ultrasounds themselves. They were asked to share their subjective assessment of the survey’s clarity. Overall, the questionnaire was deemed relevant and clear. Thus, no modifications were required before implementation. The KAP survey is shown in Table 1.

**Table 1:** Knowledge, attitude and practice (KAP) survey on the sonographic assessment of fetal head station by means of the angle of progression (AoP) and position during labor. In this quasi-experimental study with pre- and post-research design, 21 midwives working in the labor ward of the University Hospital Leuven, were recruited. The KAP survey was completed upon enrolment (n=21) and after the completion of a short online education module on these topics (n=12)

*Knowledge – sonographic assessment head station*	*Yes*	*No*	*No idea*
1) An AoP of 140° corresponds to a descent of the fetal head to the level of the ischial spines.			
2) The AoP is measured by placing the ultrasound probe horizontally on the perineum.			
3) The AoP is defined as the angle between: a) A midline through the symphysis pubis (pubic bone) b) A line from the lower edge of the symphysis pubis to the most prominent part of the fetal head in the pelvis (fetal skull).			
4) The AoP is measured by placing the ultrasound probe vertically on the perineum, on the midline.			
5) An AoP of approximately 120° means that the fetal head is located approximately at the level of the ischial spines.			
***Knowledge – sonographic assessment head position***	***Yes***	***No***	***No idea***
1) The position of the fetal head can only be determined by ultrasound via the abdomen (transabdominal sonography).			
2) The position of the fetal head can be determined by ultrasound both transabdominally and transperineally, regardless of the degree of fetal head descent.			
3) The ‘midline cerebral echo’ is an important landmark for determining the position of the fetal head.			
4) Ultrasound is generally a more reliable method for determining the position of the fetal head during labor (compared to a traditional digital vaginal examination).			
5) To determine fetal occiput position via transabdominal ultrasound, the ultrasound probe is best placed on the abdomen both in a vertical plane and a horizontal plane.			
***Attitude***	***Yes***		***No***
1) Do you believe that the use of transperineal ultrasound during labor is acceptable for parturients without epidural analgesia?			
2) Do you believe that the use of transperineal ultrasound during labor is acceptable for parturients with epidural analgesia?			
3) Do you believe that transperineal ultrasound is safe to use during labor?			
4) Do you believe that the use of ultrasound during labor (e.g. to determine fetal occiput position) can influence medical decision-making?			
5) Do you believe that the use of ultrasound during labor is time-efficient? (In other words, do you think this would allow you to organize your care more efficiently?)			
***Practice***	***Yes***		***No***
1) Do you sometimes use ultrasound yourself to determine fetal occiput position in case of slow progress or arrest during the first stage of labor?			
2) Do you sometimes use ultrasound yourself to determine fetal head station in case of slow progress or arrest during the first stage of labor?			
3) Do you sometimes use ultrasound yourself to determine fetal occiput position in case of slow progress or arrest during the second stage of labor?			
4) Do you sometimes use ultrasound yourself to determine fetal head station in case of slow progress or arrest during the second stage of labor?			

### Intervention

A baseline KAP survey was completed upon enrolment. After completion, participants were asked to read the 2018 ISUOG practice guideline^2^ and watch the corresponding video^8^ as part of an online education module, sent out through a personal link using the Research Electronic Data Capture tool (REDCap^®^ 13.1.9, Vanderbilt University, Nashville, TN, USA). Within four weeks of completion, they were asked to complete the KAP survey for a second time. Survey scores were subsequently calculated. For the knowledge section, one point was attributed to a correct answer, a score of zero to ‘no idea’ and a score of -0.5 to an incorrect answer. For the attitude and practice section, a score of one was attributed to a positive answer (i.e. in favor of intrapartum ultrasound) and a score of zero to a negative answer.

### Statistical analysis

The distribution of continuous variables was assessed based on visual inspection of histograms and as per the Kolmogorov-Smirnov test. Because of their skewed distribution, continuous variables are reported as medians and interquartile ranges (IQRs). Categorical variables are reported as absolute numbers and relative frequencies (%). To measure the effect of the online education module on their knowledge of and attitude towards intrapartum ultrasound, survey scores were compared between both timepoints using a two-sided Wilcoxon signed rank test^9^. A p< 0.05 was considered significant. Statistical analyses were conducted using SPSS (version 29.0.2.0) and Microsoft Excel (version 16.69.1).

## RESULTS

A total of 45 midwives were eligible for inclusion and invited to participate. Of these, 21 (46.7%) agreed to take part and completed the baseline KAP survey. Fifteen (33.3%) subsequently completed the online education module and of these, 12 (26.7%) completed the second KAP survey.

For the baseline knowledge section on the sonographic assessment of fetal head descent, 13 (61.9%) participants had a score ≤0/5 (1/21 with a score of -1/5). Baseline scores were higher for the sonographic assessment of fetal head position, with 12 (57.1%) participants achieving a score ≥2.5/5.

Attitude scores were overall positive, as most participating midwives (59.1%, 13/21) perceived intrapartum ultrasound as an acceptable imaging technique in parturient women without epidural analgesia. In cases with epidural analgesia, the vast majority (95.5%, 21/22) deemed this acceptable. Likewise, all participants (21/21) deemed transperineal ultrasound safe to use during labor, and 95.5% (21/22) believed intrapartum ultrasound findings could influence their decision-making regarding intrapartum interventions. Most midwives (19/21, 90.5%) also perceived it as time-efficient, i.e. stating it would allow them to organize their care more efficiently by performing it themselves.

Regarding their current practice, all participating midwives indicated they did not use ultrasound themselves for assessing fetal head position or station in case of slow progress or arrest during the first stage of labor. Only two midwives indicated they sometimes used ultrasound to assess fetal head position during the second stage (9.1%), whereas none of them used it for the assessment of head station in the second stage.

Regarding confidence levels for assessing fetal head position, out of the 21 midwives, ten gave a score of 0/10, nine gave a score 1–4/10, and two a score ≥6/10. Scores were overall lower for the sonographic assessment of fetal head station, as 15 of the 21 gave a score of 0/10, four a score 1–4/10, and two a score ≥6/10.

The median interval between completion of the first KAP survey and the online educational module was 21 days (IQR: 53 days), whereas the median interval between the latter and the second KAP survey was 4 days (IQR: 8 days). After completion of the online educational module, a significant improvement in survey scores was recorded for both the sections on fetal head station [median score: before 0/5 (IQR: 2.5), and after 2.75/5 (IQR: 1.5), p=0.01] and position [median score: before 2.5/5 (IQR: 1.9), and after 3.5/5 (IQR: 2.3), p=0.04]. No significant differences were observed in the overall attitude scores, as they remained overall positive [median score: before 4.5/5 (IQR: 1.0), and after 5.0/5 (IQR: 1.0), p=0.18].

## DISCUSSION

### Main findings

Participating midwives working in our tertiary obstetric unit perceive intrapartum ultrasound for assessing fetal head position and station as a patient-friendly, time-efficient, and safe imaging modality. The majority had little experience and thus low confidence levels for performing intrapartum ultrasounds themselves. Baseline knowledge of these topics was limited but significantly improved after completing a short online education module. Scores related to the sonographic evaluation of the fetal head station through the AoP were overall the least positive.

### Comparison to the existing literature and implications for future research

Youssef et al.^10^ conducted a short survey on physicians’ perception of ultrasound use during labor among participants of a prenatal medicine and obstetric ultrasound course in Bologna, Italy, in 2013. Most participants (66.3%) believed the sonographic variables of fetal head descent were too complex to be applied in clinical practice^10^. Although we did not formally ask this, the low confidence levels reported in our survey may indicate a similar problem. As devices are available in most labor wards, lack of knowledge, skills, and experience are possible barriers to the uptake of intrapartum ultrasound. In another study from Youssef et al.^11^, a significant improvement in obstetricians’ perception of intrapartum ultrasound was demonstrated after attending a theoretical in-person course on the topic. A practical teaching session also improved their accuracy and precision for the offline assessment of the fetal head station through the AoP^11^. Evidence from a real-world clinical setting, however, is scarce, especially among midwives. In a small interventional study by Di Pasquo et al.^12^, five midwives with no relevant experience were trained on the sonographic assessment of fetal head station through either a theoretical lecture (control group, n=3) or a lecture combined with a practical training session on a purposely designed simulator (training group, n=2). The latter resulted in significantly higher image quality scores in the evaluation phase of the study, indicating simulation training could be beneficial for midwives’ learning curve^12^.

Artificial intelligence can automate several aspects of medical imaging with limited operator intervention, including acquisition guidance and automated image analyses^13^. Ghi et al.^14^ developed an AI algorithm for the automated classification of fetal occiput position (anterior vs posterior) based on transperineal ultrasound images acquired during the 2nd stage of labor. Similar efforts have been conducted for the automated assessment of fetal head station through the AoP^15^.These automated technologies can reduce the skills and knowledge required to carry out intrapartum ultrasound, therefore facilitating the uptake by less experienced caregivers. Nonetheless, further implementation research is needed to investigate how these techniques can be implemented in real-world settings, and equally important, if they can beneficially affect patient care.

### Strengths and limitations

Strengths of our study include its novelty in terms of the target population and the scope of the questionnaire. We also acknowledge several limitations. First, the study had a single-center setup. Therefore, our results have limited generalizability. Second, since training was not mandatory, roughly only half of the midwives working in our department agreed to participate. From these, just over half completed the online education module and second KAP survey. Hence, our results are likely to be positively biased by the inclusion of midwives with a specific interest in the topic. Another limitation is the fact the second KAP survey was completed closely after the online education module. Therefore, it is uncertain whether the knowledge improvement is maintained in the long-term. We also did not collect information on possible confounders, making it difficult to ascertain if the knowledge improvement after the online education module was not influenced by other effect modifiers.

## CONCLUSIONS

Although having little experience with intrapartum ultrasound themselves, most participating midwives perceived it as an acceptable, time-efficient, and safe imaging modality. A short online education module on the sonographic assessment of fetal head position and station resulted in a significant improvement in their knowledge of these topics. Further implementation research is needed to investigate how the uptake of intrapartum ultrasound can be improved amongst midwives, and equally important, how this can improve labor care, for instance in terms of parturient satisfaction.

## Data Availability

The data supporting this research are available from the authors on reasonable request.

## References

[1] Rizzo G, Ghi T, Henrich W, et al. Ultrasound in labor: clinical practice guideline and recommendation by the WAPM-World Association of Perinatal Medicine and the PMF-Perinatal Medicine Foundation. J Perinat Med. 2022;50(8):1007-1029. doi:10.1515/jpm-2022-0160 35618672

[2] Ghi T, Eggebø T, Lees C, et al. ISUOG Practice Guidelines: intrapartum ultrasound. Ultrasound Obstet Gynecol. 2018;52(1):128-139. doi:10.1002/uog.19072 29974596

[3] Usman S, Barton H, Wilhelm-Benartzi C, Lees CC. Ultrasound is better tolerated than vaginal examination in and before labour. Aust N Z J Obstet Gynaecol. 2019;59(3):362-366. doi:10.1111/ajo.12864 30024022

[4] Chan YT, Ng KS, Yung WK, Lo TK, Lau WL, Leung WC. Is intrapartum translabial ultrasound examination painless? J Matern Fetal Neonatal Med. 2016;29(20):3276-3280. doi:10.3109/14767058.2015.1123241 26699380

[5] Seval MM, Yuce T, Kalafat E, et al. Comparison of effects of digital vaginal examination with transperineal ultrasound during labor on pain and anxiety levels: a randomized controlled trial. Ultrasound Obstet Gynecol. 2016;48(6):695-700. doi:10.1002/uog.15994 27300158

[6] Tao H, Wang R, Liu W, Zhao Y, Zou L. The value of intrapartum ultrasound in the prediction of persistent occiput posterior position: Systematic review and meta-analysis. Eur J Obstet Gynecol Reprod Biol. 2019;238:25-32. doi:10.1016/j.ejogrb.2019.04.041 31082740

[7] Bertholdt C, Morel O, Zuily S, Ambroise-Grandjean G. Manual rotation of occiput posterior or transverse positions: a systematic review and meta-analysis of randomized controlled trials. Am J Obstet Gynecol. 2022;226(6):781-793. doi:10.1016/j.ajog.2021.11.033 34800396

[8] ISUOG Practice Guidelines: intrapartum ultrasound. International Society of Ultrasound in Obstetrics and Gynecology. August 1, 2018. Accessed October 30, 2024. https://www.isuog.org/resource/isuog-practice-guidelines-intrapartum-ultrasound.html 10.1002/uog.19072 29974596

[9] Divine G, Norton HJ, Hunt R, Dienemann J. A review of analysis and sample size calculation considerations for Wilcoxon tests. Anesth Analg. 2013;117(3):699-710. doi:10.1213/ANE.0b013e31827f53d7 23456667

[10] Youssef A, Ghi T, Awad EE, et al. Ultrasound in labor: a caregiver’s perspective. Ultrasound Obstet Gynecol. 2013;41(4):469-470. doi:10.1002/uog.12267 22807171

[11] Youssef A, Kamel R. Ultrasound in labor: impact of a theoretical and practical course on caregiver’s perspective and accuracy. J Matern Fetal Neonatal Med. 2020;33(18):3163-3169. doi:10.1080/14767058.2019.1570113 30700229

[12] di Pasquo E, Ramirez Zegarra R, Kiener AJO, et al. Usefulness of an Intrapartum Ultrasound Simulator (IUSim™) for Midwife Training: Results from an RCT. Fetal Diagn Ther. 2021;48(2):120-127. doi:10.1159/000512047 33296898

[13] Drukker L, Noble JA, Papageorghiou AT. Introduction to artificial intelligence in ultrasound imaging in obstetrics and gynecology. Ultrasound Obstet Gynecol. 2020;56(4):498-505. doi:10.1002/uog.22122 32530098 PMC7702141

[14] Ghi T, Conversano F, Ramirez Zegarra R, et al. Novel artificial intelligence approach for automatic differentiation of fetal occiput anterior and non-occiput anterior positions during labor. Ultrasound Obstet Gynecol. 2022;59(1):93-99. doi:10.1002/uog.23739 34309926

[15] Conversano F, Peccarisi M, Pisani P, et al. Automatic ultrasound technique to measure angle of progression during labor. Ultrasound Obstet Gynecol. 2017;50(6):766-775. doi:10.1002/uog.17441 28233418

